# 2-Benzyl-benzofurans from the tubers of *Ophiopogon japonicus*

**DOI:** 10.1186/s13065-017-0242-z

**Published:** 2017-02-06

**Authors:** Nguyen Hai Dang, Nguyen Dinh Chung, Ha Manh Tuan, Nguyen Van Thanh, Nguyen Tuan Hiep, Dongho Lee, Nguyen Tien Dat

**Affiliations:** 1 Advanced Center for Bio-organic Chemistry, Institute of Marine Biochemistry, Vietnam Academy of Science and Technology (VAST), 18-Hoang Quoc Viet, Cau Giay, Hanoi, Vietnam; 2 Institute of Marine Biochemistry, Vietnam Academy of Science and Technology (VAST), 18-Hoang Quoc Viet, Cau Giay, Hanoi, Vietnam; 3 Graduate University of Science and Technology, Vietnam Academy of Science and Technology (VAST), 18-Hoang Quoc Viet, Cau Giay, Hanoi, Vietnam; 4 National Institute of Medicinal Materials, 1B Quang Trung, Hoan Kiem, Hanoi, Vietnam; 5 Department of Biosystems and Biotechnology, College of Life Sciences and Biotechnology, Korea University, Seoul, 02841 Republic of Korea

**Keywords:** *Ophiopogon japonicas*, Dihydrobenzofuran, 2-Benzyl-2,3-dihydroxybenzofuran, 2-Benzyl-benzofuran, Inhibition of NO production

## Abstract

**Background:**

The overproduction of nitric oxide (NO) is known to involve in various inflammatory processes. A methanol extract of the tubers of *Ophiopogon japonicus* was found to strongly inhibit NO production. The present paper deals with the isolation, structural identification and NO inhibitory effect of five compounds isolated from the MeOH extract of *O. japonicus* tubers.

**Results:**

Three new compounds were elucidated to be (2*R*)-(4-methoxybenzyl)-5,7-dimethyl-6-hydroxyl-2,3-dihydrobenzofuran (**1**), 2-(2-hydroxyl-4-methoxy-benzyl)-5-methyl-6-methoxyl-2,3-dihydrobenzofuran (**2**), and 2-(4-hydroxy-benzyl)-5,6-dihydroxybenzofuran (**3**). In addition, two known compounds were isolated from a natural source for the first time including 2-(4-methoxy-benzyl)-6,7-dimethoxyl-2,3-dihydrobenzofuran (**4**), and 2-(4-methoxy-benzyl)-6,7-methylenedioxy-2,3-dihydrobenzofuran (**5**). The absolute configuration of compound **1** was determined by experimental and calculated circular dichroism spectra. The effects of the isolated compounds on LPS-induced NO production in RAW264.7 cells were evaluated. Compound **1** and **2** showed the inhibitory activity with IC_50_ values of 11.4 and 29.1 μM, respectively.

**Conclusions:**

The class of 2-benzyl-2,3-dihydrobenzofuran is uncommon in nature. In this work, three such compounds were isolated from *O*. *japonicus*. Two of them showed promising anti-inflammatory activity by inhibition of NO production.

**Electronic supplementary material:**

The online version of this article (doi:10.1186/s13065-017-0242-z) contains supplementary material, which is available to authorized users.

## Background

*Ophiopogon japonicus* (L.f) Ker-Gawl (Convallariaceae) occurs widely in Vietnam and it has been used in traditional medicine to treat cough, fever, epistaxis, inflammation, respiratory disease, constipation, and gastrointestinal disorders [1]. Steroidal saponins are among the main characteristic components of *O*. *japonicus* and have anticancer, anti-inflammatory, antioxidative, and neuritogenic effects [2–4]. Homoisoflavonoids are also characteristic of *O*. *japonicus* and possess anti-inflammatory, antioxidative, and cytotoxic activities [4–8]. *O*. *japonicus* is also rich in polysaccharides that have antidiabetic, antioxidative, anti-inflammatory, and immunomodulatory properties [4, 7]. In addition, phenolic acids, sesquiterpenes, fatty acids, and lignans have been identified from *O*. *japonicus* [4, 9, 10].

**Fig. 1:**
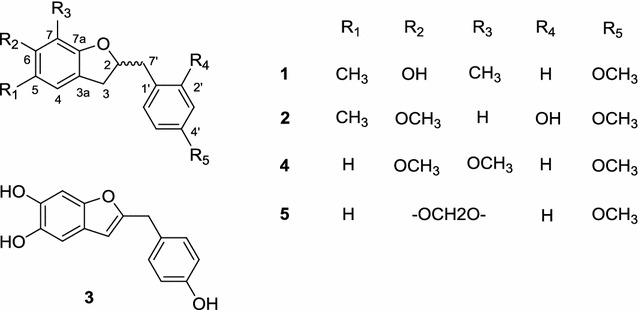
Structure of compounds **1**–**5** isolated from *O. japonicus* tubers

Nitric oxide (NO) is produced by inducible nitric oxide synthase (iNOS) in macrophages, hepatocytes, and renal cells. When produced in excess, NO directly damages normal tissues and triggers inflammation. Therefore, inhibitors of NO production have potential therapeutic value as anti-inflammatory agents [11]. In our search for anti-inflammatory compounds from natural sources, a methanol (MeOH) extract of the tubers of *O*. *japonicus* was found to strongly inhibit NO production. Phytochemical fractionation of the CHCl_3_-soluble fraction of the MeOH extract led to the isolation of five 2-benzyl benzofurans, including three new (**1**–**3**) and two known (**4**, **5**) compounds (Fig. 1). Compound **1** strongly inhibited NO production in lipopolysaccharide (LPS)-induced RAW264.7 cells.

## Results and discussion

**Fig. 2:**
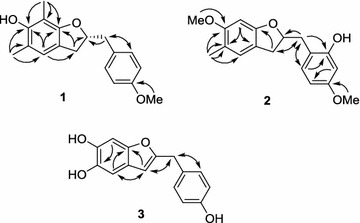
Key HMBC correlations of **1**–**3**

**Fig. 3:**
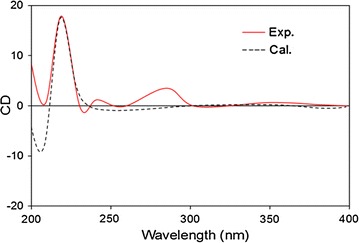
Experimental and calculated CD spectrum for compound **1**

Compound **1** was obtained as a brown solid. Its molecular formula was determined to be C_18_H_20_O_3_ from high-resolution electrospray ionisation mass spectrometry (HRESIMS) (*m*/*z* 283.1365 [M − H]^−^). Its ^1^H NMR spectrum showed the characteristic resonance of an AA′BB′ aromatic ring [δ_H_ 7.19 (2H, d, *J* = 8.5 Hz, H-2′, 6′), and 6.86 (2H, d, *J* = 8.5 Hz, H-3′, 5′)], one aromatic singlet [δ_H_ 6.67 (1H, s, H-4)], one oxygenated methine proton [δ_H_ 4.86 (partially overlapped with HDO signal, H-2)], one methoxyl group [δ_H_ 3.78 (3H, s, 4′-OMe)], two methylene groups [δ_H_ 3.07 (1H, dd, *J* = 15.0, 8.5 Hz, Ha-3), 2.82 (1H, dd, *J* = 15.0, 7.5 Hz, Hb-3), 3.02 (1H, dd, *J* = 14.0, 7.0 Hz, Ha-7′), 2.84 (1H, dd, *J* = 14.0, 6.5 Hz, Ha-7′)] and two aromatic methyl groups [δ_H_ 2.12 (3H, s, Me-5) and 2.05 (3H, s, Me-7)] (Table 1). The ^13^C NMR and DEPT spectra revealed the presence of two methyl carbons at δ_C_ 9.2 (7-Me) and 16.5 (5-Me), two methylene carbons at δ_C_ 35.9 (C-3) and 42.0 (C-7′), one methoxy carbon at δ_C_ 55.7 (4′-OMe), one oxygenated methine carbon at δ_C_ 85.1 (C-2), five methine carbons at δ_C_ 123.9 (C-4), 131.4 (C-2′, 6′), and 114.7 (C-3′, 5′), and seven quaternary carbons at δ_C_ 153.8 (C-6, observed from HMBC spectrum), 158.1 (C-7a), and 159.8 (C-4′), 118.0 (C-3a), 117.3 (C-5), 108.1 (C-7), and 131.2 (C-1′) (Table 2). These data suggested the 2-benzyl-2,3-dihydrobenzofuran skeleton of **1** [12, 13]. The HMBC correlations from aromatic singlet H-4 to C-3, C-3a, C-5, C6, C-7a, and from Me-5 to C-4, C-5, C-6, as well as from Me-7 to C-6, C-7, C-7a indicated the presence of a dihydrobenzofuran skeleton with a hydroxyl group located at C-6 and two methyl groups located at C-5 and C-7. The methoxyl group was placed on C-4′ based on the HMBC correlation of the proton of this group with C-4′ (Fig. 2). From these data, **1** was identified as 2-(4-methoxybenzyl)-5,7-dimethyl-6-hydroxyl-2,3-dihydrobenzofuran. The quantum chemical electronic circular dichroism (ECD) calculation method, based on time-dependent density functional theory (TDDFT), was used to determine of the absolute configuration at C-2 [14]. The predicted ECD patterns for 2*R* were consistent with the experimentally measured ECD of **1** (Fig. 3). Thus, compound **1** was assigned as (2*R*)-(4-methoxybenzyl)-5,7-dimethyl-6-hydroxyl-2,3-dihydrobenzofuran.

**Table 1:** ^1^H NMR data of compounds **1**–**5** (δ_H_ in ppm, *J* in Hz)

Position	**1** (CD_3_OD)	**2** (CDCl_3_)	**3** (CD_3_OD)	**4** (CDCl_3_)	**5** (CDCl_3_)
2	4.86, partially overlapped	5.05, m	–	5.00, m	5.02, m
3	3.07, dd (15.0, 8.5) 2.82, dd (15.0, 7.5)	3.15, dd (15.0, 8.5) 2.94, dd (15.0, 8.5)	6.16, s	3.12, dd (15.0, 8.0) 2.88, dd (15.0, 8.5)	3.09, dd (15.0, 8.5) 2.87, dd (15.0, 8.5)
4	6.67, s	6.87, s	6.82, s	6.74, d (8.0)	6.58, d (8.0)
5	–	–	–	6.38, d (8.0)	6.36, d (8.0)
7	–	6.39, s	6.83, s	–	–
2′	7.19, d (8.5)	–	7.10, d (8.5)	7.18, d (8.5)	7.17, d (8.5)
3′	6.86, d (8.5)	6.48, d (2.5)	6.74, d (8.5)	6.85, d (8.5)	6.85, d (8.5)
5′	6.86, d (8.5)	6.43, dd (8.0, 2.5)	6.74, d (8.5)	6.85, d (8.5)	6.85, d (8.5)
6′	7.19, d (8.5)	6.98, d (8.0)	7.10, d (8.5)	7.18, d (8.5)	7.17, d (8.5)
7′	3.02, dd (14.0, 7.0) 2.84, dd (14.0, 6.5)	3.07, dd (15.0, 3.5) 3.01, dd (15.0, 7.0)	3.91, s	3.16, dd (14.0, 6.0) 2.90, dd (14.0, 6.0)	3.13, dd (14.0, 6.0) 2.90, dd (14.0, 6.0)
5-Me	2.12, s	2.10, s	–	–	–
7-Me	2.05, s	–	–	–	–
4′-OMe	3.78, s	3.75, s	–	3.79, s	3.78, s
6-OMe	–	3.76, s	–	3.82, s	–
7-OMe	–	–	–	3.93, s	–
-OCH_2_O-	–	–	–	–	5.90, s

**Table 2:** ^13^C NMR data of compounds **1**–**5**

Position	**1** (CD_3_OD)	**2** (CDCl_3_)	**3** (CD_3_OD)	**4** (CDCl_3_)	**5** (CDCl_3_)
2	85.1	85.8	158.3	85.3	86.2
3	35.9	34.0	103.5	34.3	34.5
3a	118.0	117.2	122.0	121.2	122.9
4	123.9	126.2	106.0	118.3	116.8
5	117.3	119.0	143.1	109.9	104.3
6	153.8	157.8	144.3	152.1	148.7
7	108.1	93.6	98.6	133.7	130.0
7a	158.1	157.2	150.7	151.4	141.9
1′	131.2	116.2	130.1	129.3	129.1
2′	131.4	156.2	130.8	130.4	130.3
3′	114.7	102.9	116.2	113.9	113.9
4′	159.8	160.2	157.0	158.4	158.3
5′	114.8	106.4	116.2	113.9	113.9
6′	131.4	132.1	130.8	130.4	130.3
7′	42.0	36.9	34.8	40.9	40.7
5-Me	16.5	15.8	–	–	–
7-Me	9.2	–	–	–	–
4′-OMe	55.7	55.6	–	55.3	55.2
6-OMe	–	55.3	–	56.4	–
7-OMe	–	–	–	60.5	–
-OCH_2_O-	–	–	–	–	100.2

Compound **2** was obtained as a brown solid. Analysis of the HRESIMS spectrum indicated that compound **2** has molecular formula C_18_H_20_O_4_ (*m*/*z* 301.1436 [M + H]^+^). The ^1^H NMR spectrum of **2** showed the presence of two aromatic singlets (δ_H_ 6.87 and 6.39), an aromatic ABX spin system [δ_H_ 6.48 (1H, d, *J* = 2.5 Hz, H-3′), 6.43 (1H, dd, *J* = 8.0, 2.5 Hz, H-5′), and 6.98 (1H, d, *J* = 8.0 Hz, H-6′)], one aromatic methyl (δ_H_ 2.10) and two methoxy groups (δ_H_ 3.75 and 3.76). The ^13^C NMR and DEPT spectra of **2** showed the characteristics of 2-benzyl-2,3-dihydrobenzofuran, which are similar to those of **1** with one oxygenated methine carbon at δ_C_ 85.8 (C-2) and two methylenes at 34.0 (C-3) and 36.9 (C-7) (Table 2). The HMBC correlations confirmed the positions of the methyl and methoxy groups (Fig. 2). In a test of the optical rotation, this compound was optically inactive. Therefore, the structure of compound **2** was determined to be 2-(2-hydroxyl-4-methoxy-benzyl)-5-methyl-6-methoxyl-2,3-dihydrobenzofuran.

Compound **3** was obtained as a brown solid. Its HRESIMS revealed a molecular ion peak at *m*/*z* 255.0632 [M − H]^−^ corresponding to a molecular formula of C_15_H_12_O_4_. The ^1^H NMR spectrum showed signals due to an olefinic proton [δ_H_ 6.16 (1H, s)], two aromatic protons [δ_H_ 6.82 (1H, s), and 6.83 (1H, s)], a 1,4-disubstituted benzene ring with two apparent doublets [δ_H_ 7.10 (2H, d, *J* = 8.5 Hz), 6.74 (2H, d, *J* = 8.5 Hz)], and a methylene group [δ_H_ 3.91 (2H, s)]. The ^13^C NMR and DEPT experiments showed one methylene, seven methines, five oxygenated aromatic carbons, and two quaternary carbons. In the HMBC spectrum, the correlations between the olefinic proton (δ_H_ 6.16, H-3) and C-3a (δ_C_ 122.0), C-4 (δ_C_ 106.0), and C-7a (δ_C_ 150.7), H-4/C-3 (δ_C_ 103.5), C-3a, C-5 (δ_C_ 143.1), C-6 (δ_C_ 144.3), and C-7a, as well as H-7/C-3a, C-5, C-6, and C-7a indicated the presence of a benzofuran skeleton with two oxygen substituents on ring A (Fig. 2). Similarly, the correlations between the 1,4-disubstituted protons (δ_H_ 7.10, H-2′,6′) and the oxygenated aromatic carbon (δ_C_ 157.0) and the methylene carbon (δ_C_ 34.8) indicated the presence of a 4-hydroxybenzyl group. Moreover, the HMBC correlations of the methylene protons (δ_H_ 3.19, H-7′) with C-2, C-3, and C-4 suggested the location of the 4-hydroxy-benzyl group at C-2 (Additional file 1). This compound was also optically inactive. Therefore, the structure of **3** was assigned as 2-(4-hydroxy-benzyl)-5,6-dihydroxybenzofuran.

Compounds **4** and **5** were identified as 2-(4-methoxy-benzyl)-6,7-dimethoxyl-2,3-dihydrobenzofuran, and 2-(4-methoxy-benzyl)-6,7-methylenedioxy-2,3-dihydrobenzofuran, respectively [12]. Both compounds were isolated for the first time from the nature. It is noted that the class of 2-benzyl-2,3-dihydrobenzofuran is uncommon and only a few compounds of this type have been isolated so far from natural source [15].

The isolated compounds were tested for their ability to inhibit NO production in LPS-stimulated RAW264.7 cells. NO is an important signalling molecule in various physiological and pathophysiological responses, including the circulation, blood pressure, platelet function, and host defence. The overproduction of NO is also important in inflammation and related processes [16]. High NO levels are used as a marker for the treatment of inflammatory disorders. According to its specific functions and characteristics, inhibition of NO production by immune cells, typically macrophages, is suggested as one strategy for the development of anti-inflammatory agents. Our test revealed that compounds **1** and **2** reduced NO levels in LPS-stimulated RAW264.7 cells. Compound **1** was the most active compound with an IC_50_ of 11.4 μM, while compound **2** had a moderate effect (IC_50_ = 29.1 μM). Compounds **3**–**5** were inactive up to the highest concentration tested (30 μM). The cell viability test showed that neither compound had significant toxicity at its effective dose for NO inhibition (data not shown). 2,3-Dihydrobenzofurans have been known as potent anti-inflammatory compounds. Closse et al. [17] demonstrated that the synthetic analogues of 2,3-dihydrobenzofuran-2-one had powerful anti-inflammatory activity in vivo, and 5-chloro-6-cyclohexyl-2,3-dihydrobenzofuran-2-one was significantly more potent than the reference compound, diclofenac, in all testing models. More recently, a series of dihydrobenzofurans was isolated from the seeds of *Prunus tomentosa*, some of which strongly inhibited NO production in LPS-stimulated BV-2 cells [18]. Consistently, our results suggest that *O*. *japonicus* is a potential natural source of anti-inflammatory dihydrobenzofurans.

## Methods

### General experimental procedures

Optical rotation values were recorded on a JASCO P-2000 digital polarimeter (JASCO, Tokyo, Japan). The IR spectra were obtained from a Tensor 37 FT-IR spectrometer (Bruker, Ettlingen, Germany). CD spectra were obtained with a JASCO J-1100 spectropolarimeter. NMR experiments were carried out on a Bruker AM500 FT-NMR spectrometer (Bruker, Rheinstetten, Germany) using residual solvent peak as a reference or tetramethylsilane (TMS) as internal standard. The HR-ESI-MS were recorded on a Waters Q-TOF micromass spectrometer Waters Q-TOF micromass spectrometer and an LTQ Orbitrap XL™ Mass spectrometer. Absorbance of bioassay solutions was read on an xMark microplate spectrophotometer.

### Plant materials

The tubers of *O. japonicus* were collected in Feb. 2014 at Me Linh, Hanoi and identified by Prof. Tran Huy Thai, Institute of Ecology and Biological Resources, Vietnam Academy of Science and Technology. The voucher specimens were deposited at the Department of Bioactive Products, Institute of Marine Biochemistry, Vietnam Academy of Science and Technology.

### Extraction and isolation

The air-dried and powdered tubers of *O. japonicus* (2.4 kg) were extracted with methanol (4 L × 3 times) in a sonic bath for 30 min at 40 °C. The combined extracts were concentrated under a vacuum to obtain a crude residue (360 g), which was then resuspended in water (2 L), and extracted by chloroform (1 L × 3 times) to obtain chloroform (8 g) and water residues. The chloroform residue was chromatographed on a silica gel column eluted with a gradient of 1–100% ethyl acetate in hexane to afford nine fractions F1–F9. Fraction F1 was fractionated on a silica gel column eluted with hexane–ethyl acetate (20:1 v/v) to give nine subfractions F1.1–F1.9. Compound **5** (69.5 mg) was purified from F1.4 by using a reverse phase C_18_ column eluted with acetone–water (2:1 v/v). Compound **1** (70.0 mg) and **4** (18.2 mg) were isolated from F1.7 by using a reverse phase C_18_ column eluted with acetone–water (3:2 v/v). The F1.9 was chromatographed on a silica gel column (hexane–acetone 8:1, v/v) to afford **2** (20.8 mg). Compound **3** (20.0 mg) was obtained from F9 by repeated C_18_ column (methanol–water 1:1 v/v) and silica gel column (dichloromethane-acetone 6:1, v/v).

### (2R)-(4-methoxybenzyl)-5,7-dimethyl-6-hydroxyl-2,3-dihydrobenzofuran (1)

Brown solid. [α]_D_^24^ = + 86.5 (*c* 0.05, MeOH). IR (KBr): 3446, 1615, 1513, 1472, 1247, 1097. CD (MeOH): 220 (+17.8), 288 (+3.2). ^1^H-(CD_3_OD, 500 MHz) and ^13^C-NMR (CD_3_OD, 125 MHz): see the Tables 1 and 2. HR-ESI-MS (neg.): 283.1365 [M − H]^−^ (calc. for C_18_H_19_O_3_, 283.1334).

### 2-(2-Hydroxyl-4-methoxy-benzyl)-5-methyl-6-methoxyl-2,3-dihydrobenzofuran (2)

Brown solid. IR (KBr): 3440, 1610, 1482, 1245, 1120. ^1^H-(CDCl_3_, 500 MHz) and ^13^C-NMR (CDCl_3_, 125 MHz): see the Tables 1 and 2. HR-ESI-MS (pos.): 301.1436 [M + H]^+^ (calc. for C_18_H_21_O_4_, 301.1440).

### 2-(4-Hydroxy-benzyl)-5,6-dihydroxybenzofuran (3)

Brown solid. IR (KBr): 3450, 1613, 1514, 1484, 1242, 1142. ^1^H-(CD_3_OD, 500 MHz) and ^13^C-NMR (CD_3_OD, 125 MHz): see the Tables 1 and 2. HR-ESI-MS (neg.): 255.0632 [M − H]^−^ (calc. for C_15_H_11_O_4_, 255.0657).

### Assay for inhibition of NO production

The effects of compounds on the NO production in LPS-stimulated RAW264.7 cells were evaluated as previously described [19]. The cells were seeded in 96-well plate at 2 × 10^5^ cells/well and incubated for 12 h. The plate were pretreated with compounds in various concentrations (from 1 to 30 µM) for 30 min and then incubated for another 24 h with or without 1 μg/ml LPS. 100 μl of the culture supernatant were transferred to other 96-well plate and 100 μl of Griess reagent were added. The absorbance of the reaction solution was read at 570 nm with a microplate reader (XMark microplate reader, Biorad, USA). The remaining cell solutions in cultured 96-well plate were used to evaluate cell viability by 3-(4,5-dimethylthiazole-2-yl)-2,5-diphenyl tetrazolium bromide (MTT) assay. Cardamonin was used as a positive control (IC_50_ = 2.80 μM).

## Conclusion

In summary, three 2-benzyl-2,3-dihydrobenzofurans and two 2-benzyl-benzofurans were isolated from the CHCl_3_-soluble fraction of the MeOH extract of *O*. *japonicus* tubers. Their structures were found to be (2*R*)-(4-methoxybenzyl)-5,7-dimethyl-6-hydroxyl-2,3-dihydrobenzofuran (**1**), 2-(2-hydroxyl-4-methoxy-benzyl)-5-methyl-6-methoxyl-2,3-dihydrobenzofuran (**2**), 2-(4-hydroxy-benzyl)-5,6-dihydroxybenzofuran (**3**), 2-(4-methoxy-benzyl)-6,7-dimethoxyl-2,3-dihydrobenzofuran (**4**), and 2-(4-methoxy-benzyl)-6,7-methylenedioxy-2,3-dihydrobenzofuran (**5**). Compounds **1** and **2** inhibited NO production in LPS-stimulated RAW264.7 cells.

## Additional file

**Additional file 1.** NMR spectra of compounds **1**–**3**.
