# Supplementary material for: 2-Benzyl-benzofurans from the tubers of Ophiopogon japonicus
Source: Chem Cent J. 2017 Feb 6;11:15. doi: 10.1186/s13065-017-0242-z (PMC5293711; doi:10.1186/s13065-017-0242-z)
Supplement: Supplementary file 1 — Additional file 1. NMR spectra of compounds 1–3. [file 13065_2017_242_MOESM1_ESM.docx]

Supplementary Material

2-Benzyl Benzofurans from the Tubers of *Ophiopogon japonicus*

**Nguyen Hai Dang^1^*, Nguyen Dinh Chung^2,3^, Ha Manh Tuan^2^, Nguyen Van Thanh^2,3^, Nguyen Tuan Hiep^4^, Dongho Lee^5^, Nguyen Tien Dat^2,3^***

^1^Advanced Center for Bio-organic Chemistry, Institute of Marine Biochemistry, Vietnam Academy of Science and Technology (VAST), 18-Hoang Quoc Viet, Cau Giay, Hanoi, Vietnam

^2^ Institute of Marine Biochemistry, VAST, 18-Hoang Quoc Viet, Cau Giay, Hanoi, Vietnam

^3^ Graduate University of Science and Technology, VAST, 18-Hoang Quoc Viet, Cau Giay, Hanoi, Vietnam

^4^ National Institute of Medicinal Materials, 1B Quang Trung, Hoan Kiem, Hanoi, Vietnam

^5^ Department of Biosystems and Biotechnology, College of Life Sciences and Biotechnology, Korea University, Seoul 02841, Republic of Korea

*Correspondence: [ngtiend@imbc.vast.vn](mailto:ngtiend@imbc.vast.vn) (NTD), [nguyenhd@imbc.vast.vn](mailto:nguyenhd@imbc.vast.vn) (NHD)

**Content**

Figure S1. ^1^H-NMR spectrum of **1**.

Figure S2. ^13^C-NMR spectrum of **1**.

Figure S3. DEPT spectrum of **1.**

Figure S4. HSQC spectrum of **1**.

Figure S5. HMBC spectrum of **1**.

Figure S6. NOESY spectrum of **1**.

Figure S7. ^1^H-NMR spectrum of **2**.

Figure S8. ^13^C-NMR spectrum of **2**.

Figure S9. DEPT spectrum of **2.**

Figure S10. HSQC spectrum of **2**.

Figure S11. HMBC spectrum of **2**.

Figure S12. ^1^H-NMR spectrum of **3**.

Figure S13. ^13^C-NMR spectrum of **3**.

Figure S14. DEPT spectrum of **3.**

Figure S15. HSQC spectrum of **3**.

Figure S16. HMBC spectrum of **3**.


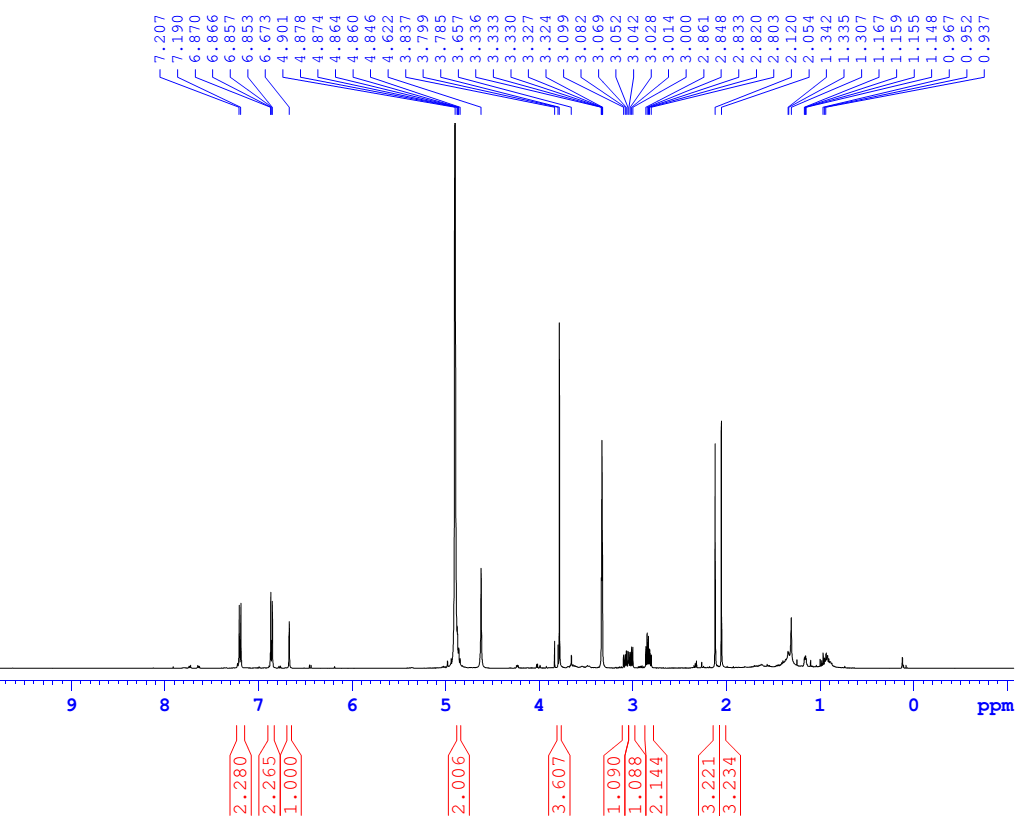


Figure S1. ^1^H-NMR spectrum of **1**.


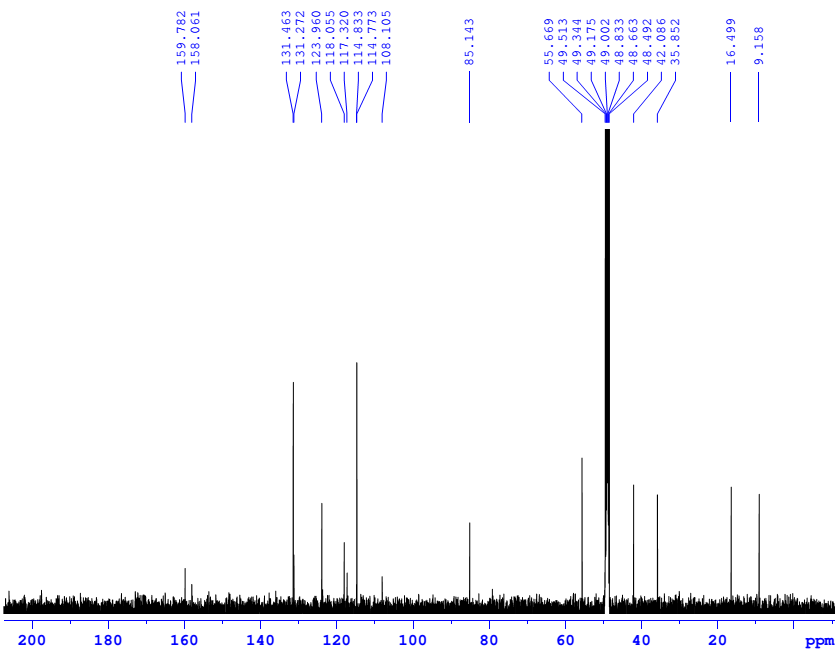


Figure S2. ^13^C-NMR spectrum of **1**.


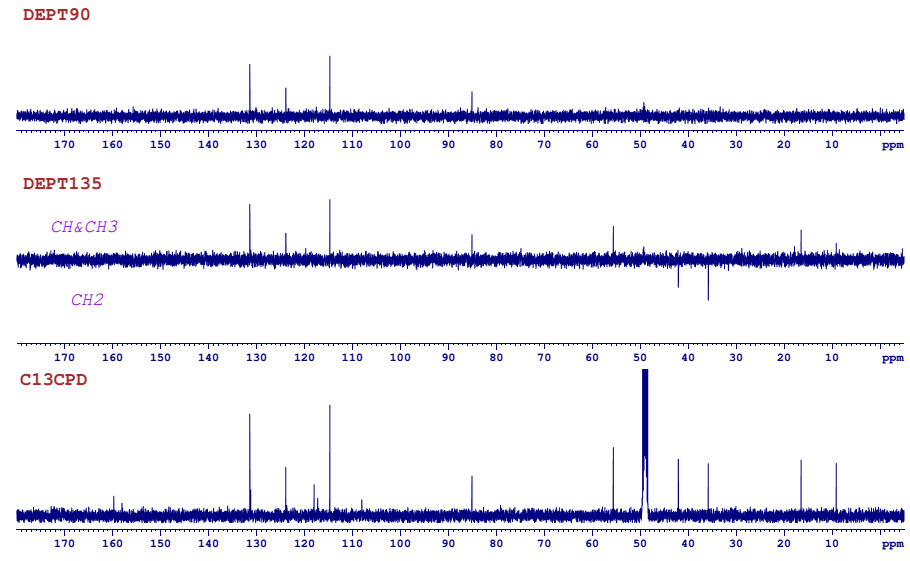


Figure S3. DEPT spectrum of **1**.


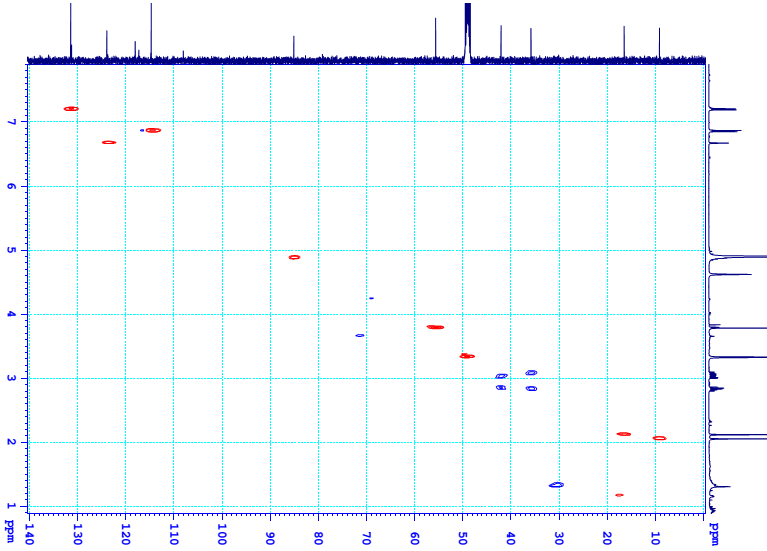


Figure S4. HSQC spectrum of **1**.


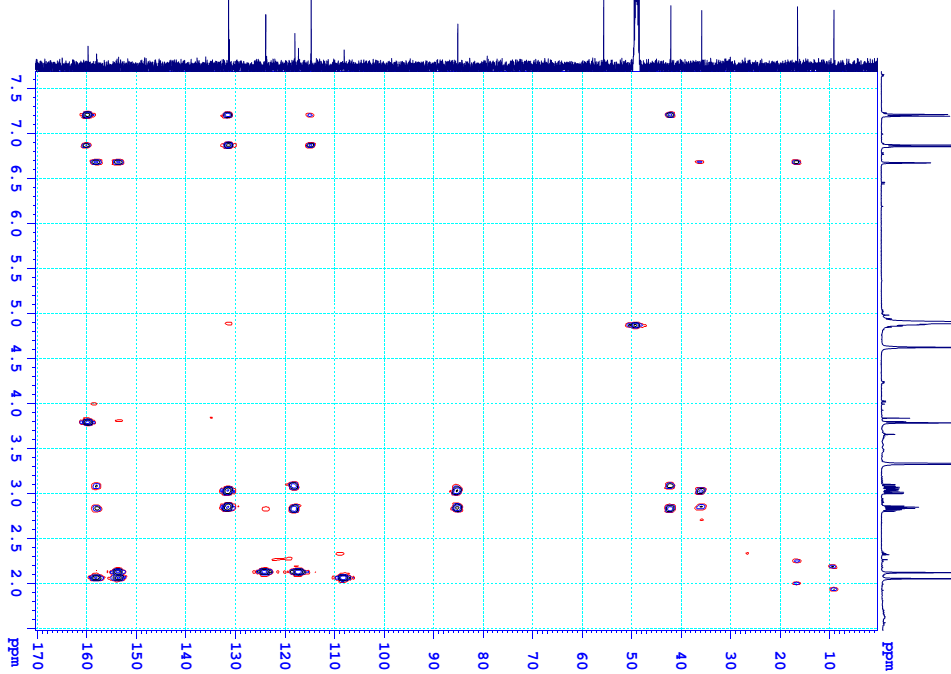


Figure S5. HMBC spectrum of **1**.


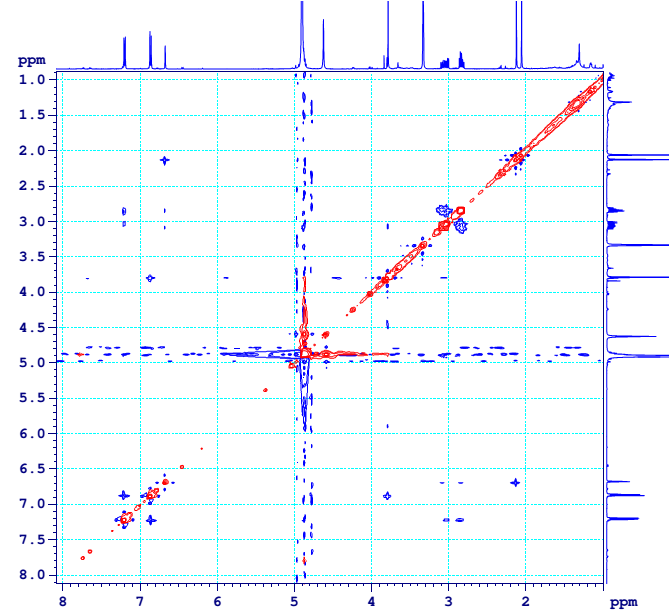


Figure S6. NOESY spectrum of **1**.


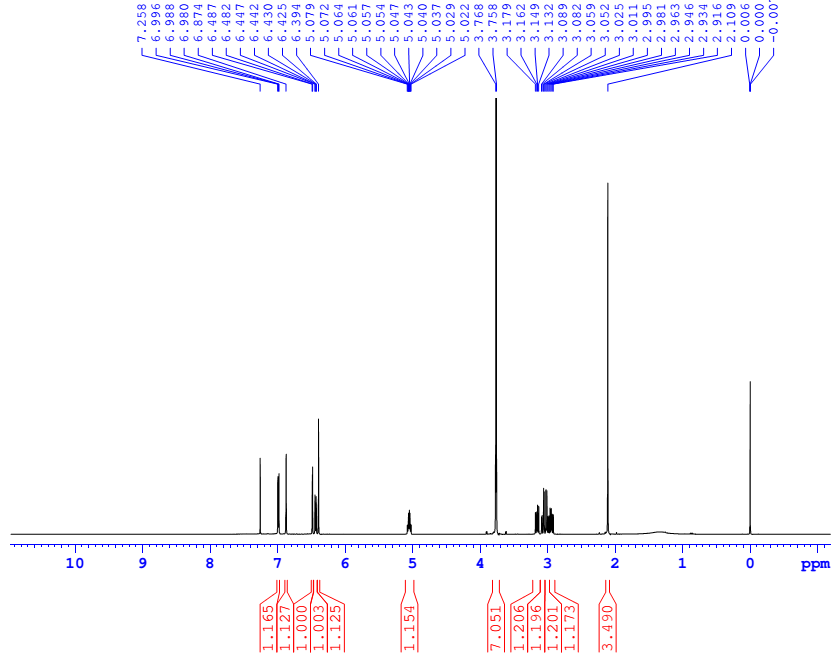


Figure S7. ^1^H-NMR spectrum of **2**.


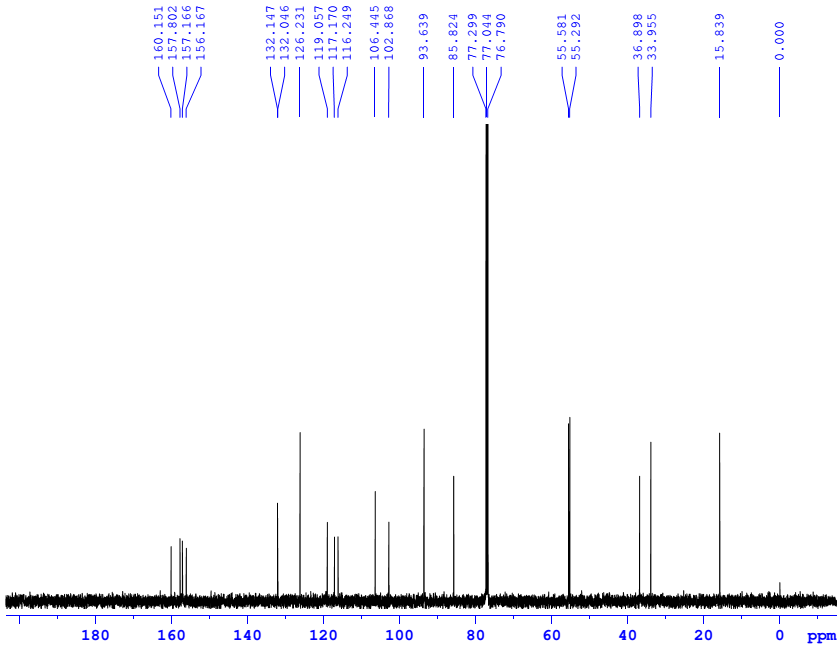


Figure S8. ^13^C-NMR spectrum of **2**.


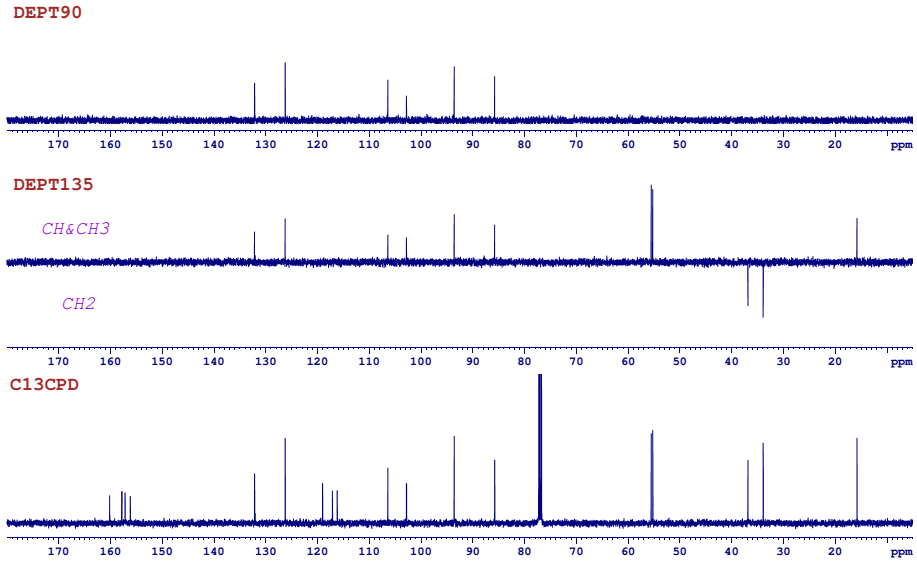


Figure S9. DEPT spectrum of **2**.


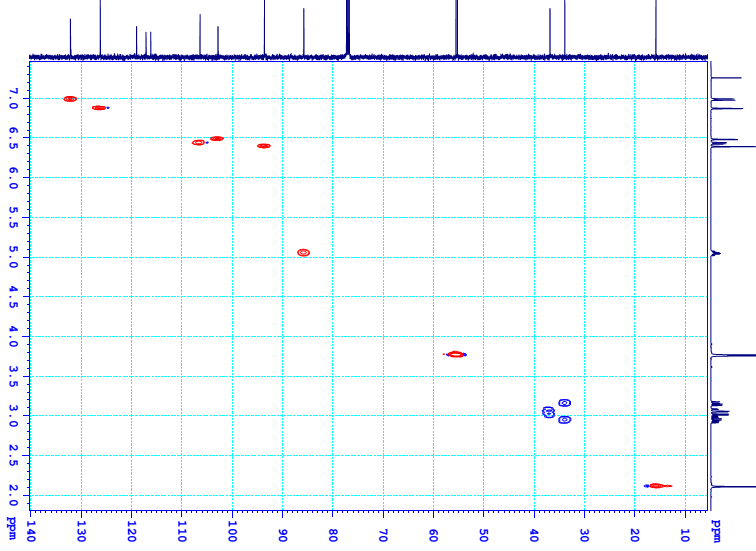


Figure S10. HSQC spectrum of **2**.


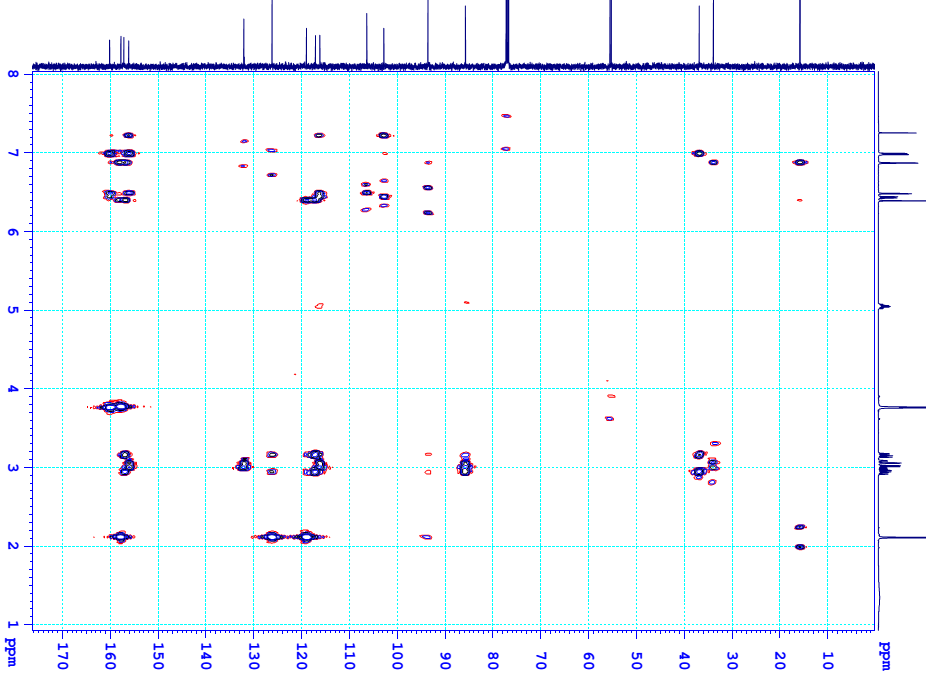


Figure S11. HMBC spectrum of **2**.


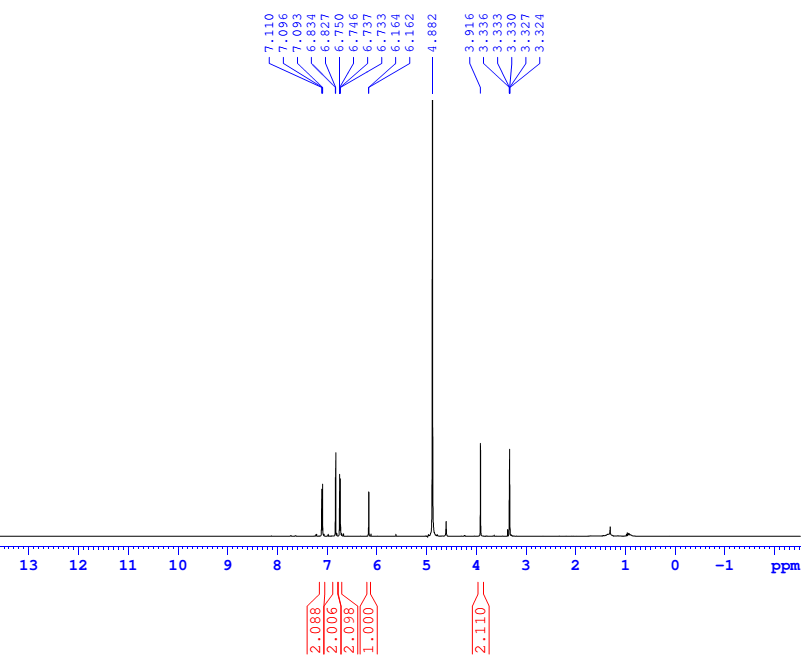


Figure S12. ^1^H-NMR spectrum of **3**.


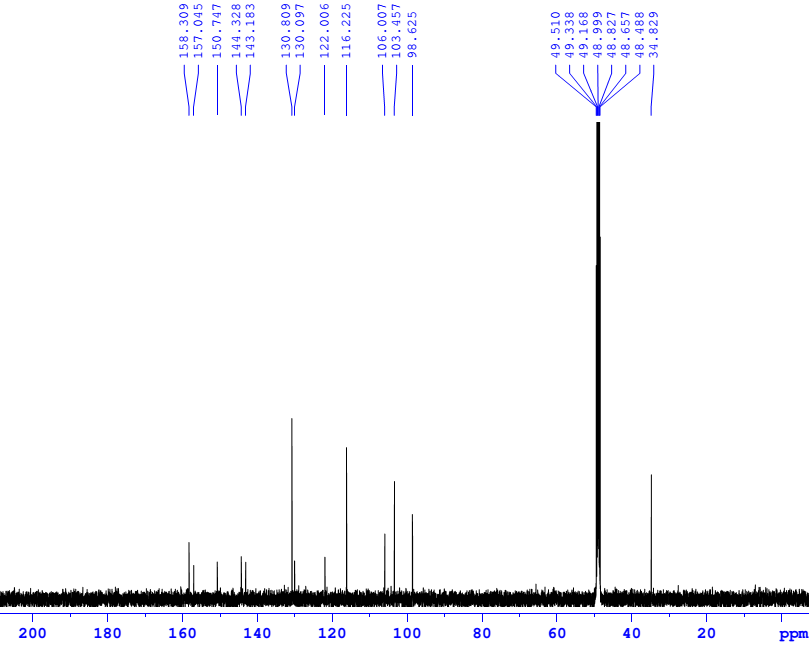


Figure S13. ^13^C-NMR spectrum of **3**.


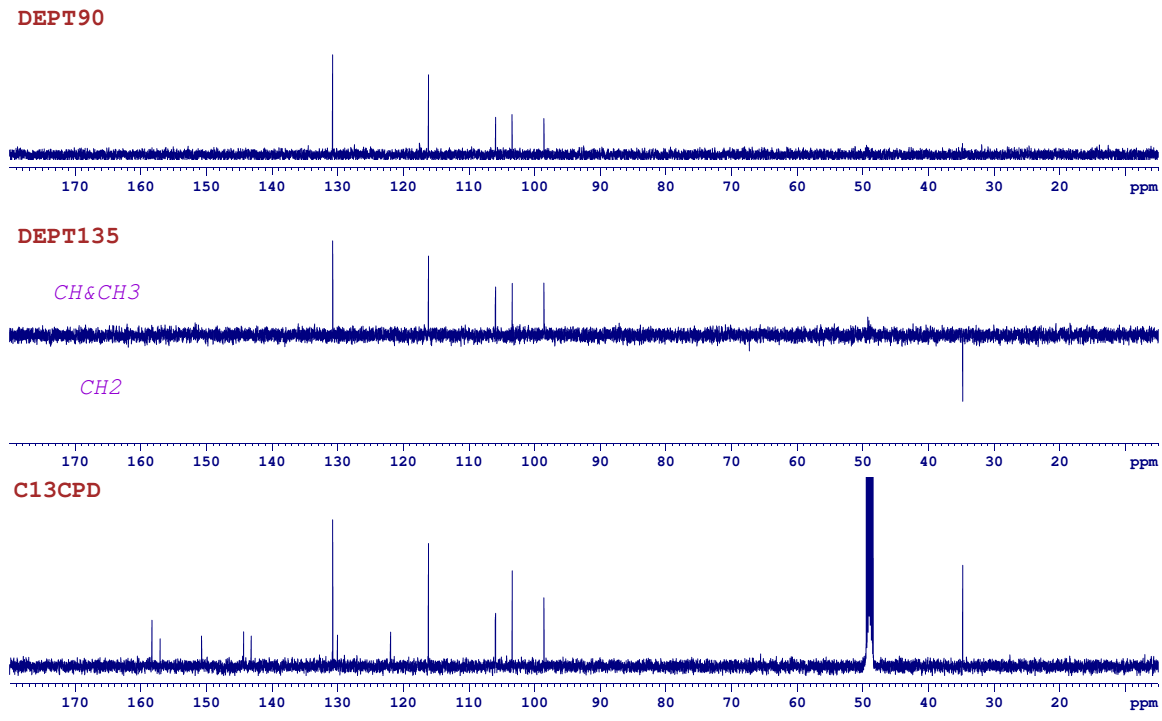


Figure S14. DEPT spectrum of **3**.


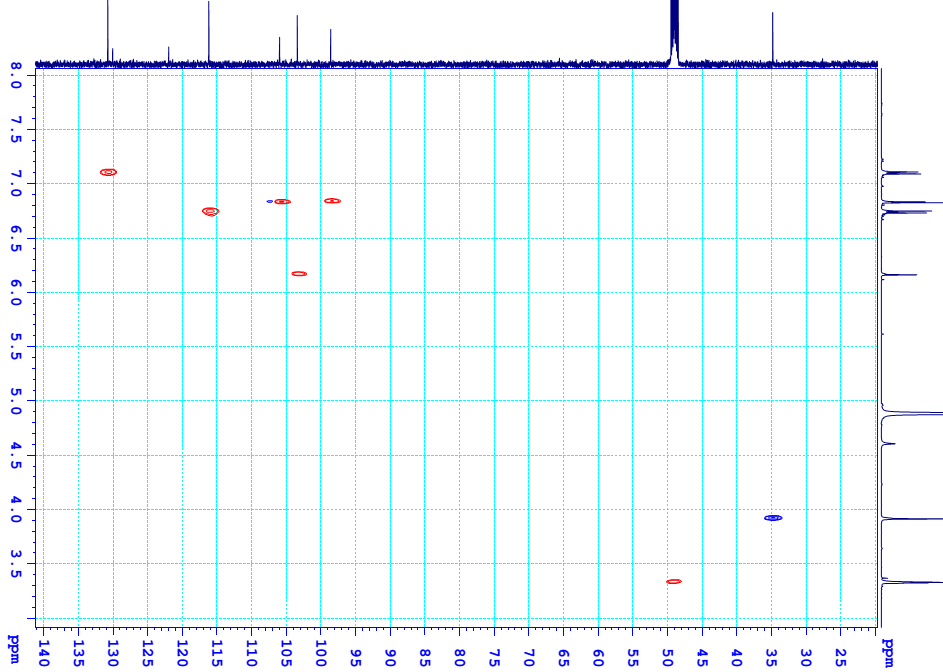


Figure S15. HSQC spectrum of **3**.


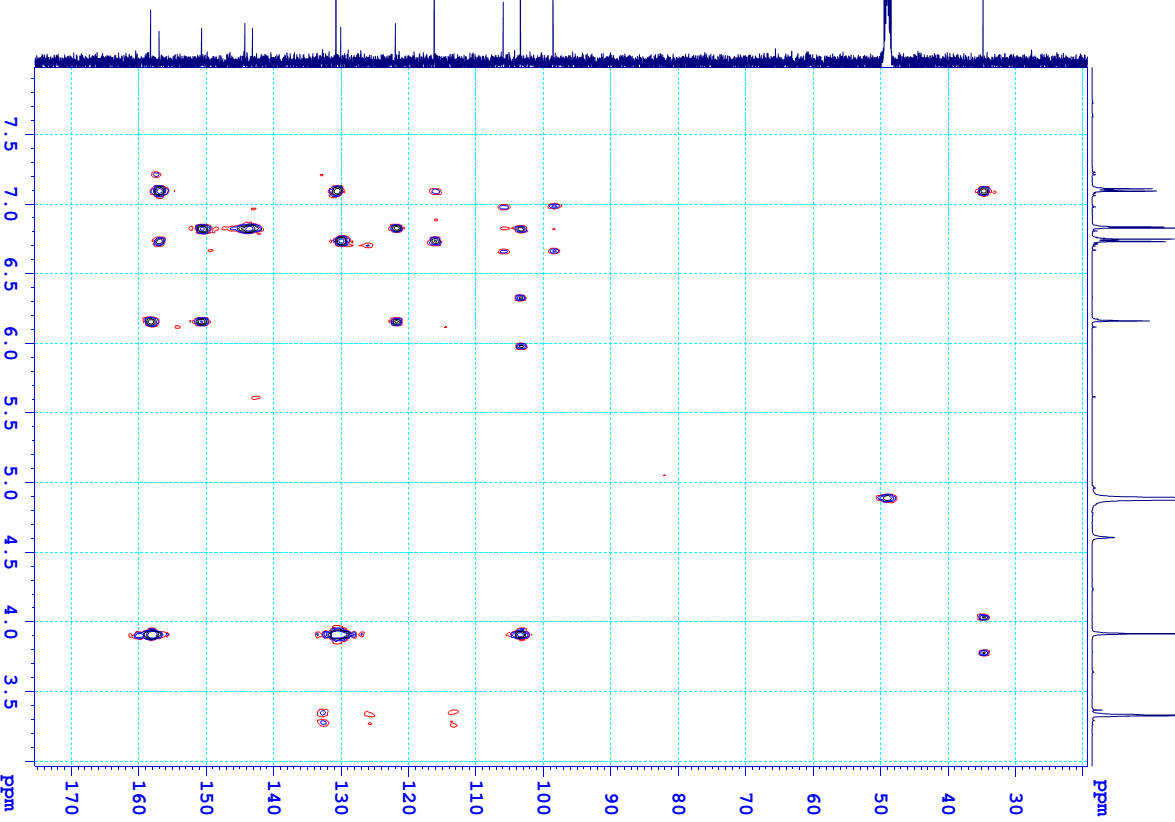


Figure S16. HMBC spectrum of **3**.
